# Optic Pathway Glioma: Current Treatment Approaches and Ongoing Clinical Trials

**DOI:** 10.3390/brainsci15080894

**Published:** 2025-08-21

**Authors:** Osama Elzaafarany, Sarah Elhomosany, Alexandra Rincones, Vincent Dlugi, Sepideh Mokhtari

**Affiliations:** 1 Neuro-Oncology, Moffitt Cancer Center, Tampa, FL 33612, USAsepideh.mokhtari@moffitt.org (S.M.); 2 Department of Ophthalmology, Faculty of Medicine, Alexandria University, Alexandria 5424041, Egypt; 3 Department of Neurology, University of South Florida, Tampa, FL 33620, USA; arincones@usf.edu

**Keywords:** neurology, ophthalmology, oncology, MEK inhibitors, clinical trials, brain/orbit MRI, optic pathway glioma, neurofibromatosis type 1

## Abstract

Optic pathway glioma (OPG) is a rare pediatric low-grade glioma, frequently associated with neurofibromatosis type 1 (NF–1), that presents unique therapeutic challenges due to its anatomical location and its potential to impair vision, endocrine function, and developmental trajectories. Current clinical management prioritizes a multidisciplinary, patient-specific approach aimed at tumor control while preserving long-term quality of life. Strategies vary based on clinical presentation, ranging from observation in asymptomatic cases to chemotherapy for progressive or symptomatic tumors. Surgical and radiation options are limited due to potential risks and complications. In recent years, advances in molecular characterization have guided the development of targeted therapies, particularly MEK inhibitors, which demonstrate encouraging efficacy and reduced toxicity profiles. In parallel, investigational therapies including immunotherapy and precision medicine-based approaches are under clinical evaluation. This review provides a synthesis of current standard practices, emerging targeted treatments, and ongoing clinical trials, drawing on relevant literature and expert consensus to inform clinicians and families about available therapeutic options. Literature discussed in this review was identified through a non-systematic search of published articles, clinical trial registries, and authoritative guidelines, with selection based on relevance, clinical significance, and contribution to understanding current and emerging management strategies for OPG.

## 1. Introduction and Background

Optic pathway gliomas (OPGs) are rare, low-grade astrocytic tumors that occur in the optic nerve, chiasm, or both, most commonly affecting children under 10 years old. Most cases are associated with neurofibromatosis type 1 (NF–1), an autosomal-dominant genetic disorder caused by mutations in the NF-1 gene, leading to abnormal regulation of cell growth. NF–1 is characterized by skin manifestations (e.g., café-au-lait spots, neurofibromas), learning difficulties, and predisposition to tumors such as OPGs [1]. While many OPGs are indolent and can remain stable without immediate intervention, others may cause significant morbidity, particularly through visual impairment, proptosis, or even neurological decline [2]. Managing OPG poses a unique challenge, requiring a delicate balance between tumor control and preservation of vision and quality of life.

Treating OPG is influenced by factors such as tumor size, growth rate, patient age, tumor location in the optic pathway, visual status, and the presence of an NF–1 diagnosis. Traditional management strategies include observation for asymptomatic or stable tumors, chemotherapy for symptomatic cases, and surgery or radiation for more aggressive lesions. Chemotherapy protocols like carbo-vincristine vinblastine single agent; and thioguanine, procarbazine, lomustine (CCNU), and vincristine (TPCV) protocols have proven effective in stabilizing or reducing tumor size, particularly for pediatric patients [3,4]. Surgical resection is reserved for certain indications but carries risks of vision loss and neurological complications [5]. Radiation therapy (RT) is avoided for young children, particularly those younger than 5 years old, because of its long-term developmental effects. RT is also avoided in NF-1 patients due to the risk of secondary malignancies in this patient population [6].

Despite these options, treating OPG remains challenging because of issues such as treatment resistance, side effects, and long-term complications. Advances in molecular genetics and targeted therapies offer hope for more effective, personalized approaches [7]. Ongoing clinical trials are exploring innovative treatments, including new drug combinations, targeted agents, and immunotherapies, aiming to improve tumor control, minimize side effects, and enhance long-term outcomes. This review highlights the current treatment landscape and emerging therapeutic options, focusing on clinical trials that represent the future of OPG management.

A version of this manuscript was previously posted as a preprint on Research Square on 27 June 2025 [8]. 

## 2. Review

### 2.1. Current Treatment Approaches for OPG

**Figure 1:**
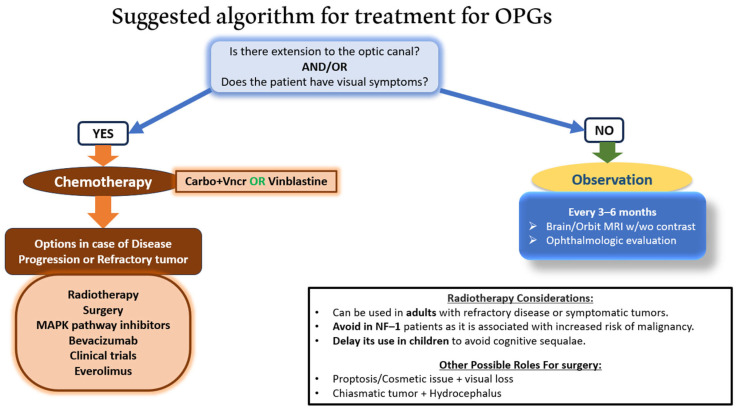
**Suggested algorithm for treatment for OPGs.** This flowchart illustrates a proposed treatment algorithm for treatment of OPGs based on the presence of optic canal extension and/or visual symptoms. If either is present, initial management with chemotherapy (carboplatin + vincristine or vinblastine) is recommended. In the case of disease progression or refractory tumors, further options include radiotherapy, surgery, MAPK pathway inhibitors, bevacizumab, clinical trials, or everolimus. Observation is advised in asymptomatic patients without optic canal involvement. The role of radiotherapy and surgery is context-dependent, with specific considerations for age and NF–1 status. Source: Figure created by the authors. Original work. Not reproduced from any external source. Abbreviations: NF–1, neurofibromatosis type 1; OPGs, optical pathway gliomas.

The current treatment options for OPG include observation, chemotherapy, RT, and surgery. Treatment decisions should be made by a multidisciplinary team, including pediatric oncologists, neuro-oncologists, neurosurgeons, ophthalmologists, and radiation oncologists, to ensure comprehensive care and minimize long-term adverse effects. Screening for NF–1 is essential for patients diagnosed with OPG. Figure 1 shows a suggested algorithm for treating OPGs.

#### 2.1.1. Observation

Observation is reserved for patients who are asymptomatic or have mild symptoms, especially for patients with NF–1. Observation may be recommended to avoid treatment-related morbidity. This approach is often chosen when the tumor is not causing immediate threats to vision or other neurological functions.

Scheduling observation visits is advised every 3 to 6 months. These visits usually involve clinical assessments and imaging tests, such as magnetic resonance imaging (MRI), to monitor for any changes in the tumor that could require treatment. The specific frequency of visits may differ based on the patient’s individual circumstances and the judgment of the treating physician. Additionally, regular ophthalmological assessments are an integral part of observation for early detection of visual changes.

The natural course of OPG among children is highly unpredictable. While some tumors remain stable for years or even regress spontaneously, other tumors grow progressively. Active treatment is typically reserved for patients experiencing progressive vision loss or those with existing visual impairment who are at high risk of further deterioration, regardless of substantial tumor progression on MRI. Tumor growth alone is not a criterion for initiating therapy. Nicolin et al. reported that approximately 48% of patients do not require immediate treatment. However, these patients still need regular neuroradiological and ophthalmological monitoring, with intervals determined by factors such as tumor location, symptoms, and the presence or absence of NF–1 association [9,10].

#### 2.1.2. Chemotherapy

Chemotherapy is typically the first-line treatment for symptomatic OPG, particularly for young children. Although many OPGs appear benign histologically, chemotherapy surprisingly yields high response rates. Consequently, chemotherapy is frequently chosen as the initial treatment over radiation, particularly for children under 5 years old, as radiotherapy can significantly harm cognitive development. By using chemotherapy, the need for radiation can often be delayed, which may lessen neurocognitive side effects without compromising the survival rates [11].

The most-used first-line chemotherapy regimen available for treating OPG is the combination of vincristine and carboplatin, which is the preferred first-line therapy, achieving a 3-year progression-free survival (PFS) rate of 77% and a 5-year PFS rate of 69% [12]. Vinblastine, as a single agent, is widely used as a first-line chemotherapy protocol in Canada, based on a phase II study from The Hospital for Sick Children in Toronto, which supports weekly vinblastine as an effective first-line or salvage monotherapy for pediatric low-grade gliomas; this approach has been broadly adopted across Canadian centers [13].

A multicenter study led by Institut Gustave Roussy in France, involving 85 children with progressive OPGs (median age, 33 months), demonstrated the effectiveness of using chemotherapy as an initial treatment to delay the need for RT. The children were treated with alternating chemotherapy regimens every 3 weeks, such as procarbazine and carboplatin, etoposide and cisplatin, or vincristine and cyclophosphamide. This strategy successfully deferred the need for RT among 75% of cases at 3 years old and 61% at 5 years old. For the 25 children who eventually required RT, the median interval from the start of treatment to the commencement of RT was 35 months. Postponing RT did not adversely affect outcomes, as the 5-year overall survival rate was 89%. This result of postponed radiation was similar to that achieved with initial radiation, and visual results were comparable [14].

Between 40% and 60% of patients eventually progress after chemotherapy and require further salvage therapies [15]. These may include RT, bevacizumab with or without irinotecan, BRAF and/or MEK inhibitors, immunotherapy, and participation in clinical trials.

The benefits of chemotherapy come with significant risks, as its side effects can be serious, including severe bone marrow suppression. Table 1 shows the most commonly used chemotherapy regimen in treating OPG, including the dosage and potential side effects.

#### 2.1.3. Radiation Therapy

Several studies have investigated the role of radiation therapy in the management of low-grade gliomas, including optic pathway gliomas (OPGs). The typical radiation dose ranges from 45 Gy to 54 Gy, delivered in fractions of 1.8 to 2 Gy. Current recommendations suggest reserving radiation therapy for children older than 8–10 years or for those with tumors resistant to chemotherapy, due to the potential for long-term complications such as cognitive impairment, secondary malignancies, and vascular injury. Additionally, radiation therapy is generally avoided in patients with neurofibromatosis type 1 (NF–1) because of the increased risk of secondary malignancies [11,16].

Radiation therapy can halt the progression of visual decline, though the full impact of RT may take years to manifest. In a retrospective analysis involving 42 patients with OPG, with a median age of 6.6 years (1.25–19 years), 29 patients received radiation because of disease progression. Tumor reduction was noted for 18% of patients at 24 months and 46% at 60 months, with a median response time of 62 months. Additionally, vision stabilization or improvement was achieved in 81% of cases. The rate of freedom from disease progression at 10 years was 89%, and the rate of overall survival at 10 years was 100% [17].

#### 2.1.4. Surgery

Surgery is not commonly used as a primary treatment but may be considered for specific cases, such as patients with single-nerve involvement causing progressive proptosis and blindness, patients with exophytic tumors involving the optic chiasm that cause mass effect or hydrocephalus. Furthermore, surgical debulking may be performed to relieve symptoms or improve vision, but complete resection is rarely possible without significant neurological injury [18].

### 2.2. Promising Treatment Options

#### 2.2.1. Mitogen-Activated Protein Kinase (MAPK) Pathway Inhibitors

Targeting the MAPK pathway has emerged as a promising strategy for treating OPG; however, the optimal timing for its implication remains controversial; some pediatric oncologists advocate for using it as a first-line therapy, while others recommend it after failure of chemotherapy or RT. Therefore, participation in clinical trials is highly encouraged [19].

The mitogen-activated protein kinase (MAPK) signaling pathway is a key intracellular cascade that regulates cell proliferation, differentiation, and survival. In its canonical form, activation begins when extracellular growth factors bind to receptor tyrosine kinases (RTKs), leading to recruitment of adaptor proteins and activation of Ras GTPases. Activated Ras triggers phosphorylation and activation of RAF kinases, which in turn phosphorylate MEK1/MEK2, leading to activation of extracellular signal-regulated kinases (ERK1/ERK2). These kinases translocate to the nucleus to regulate gene expression that promotes cell cycle progression and survival. In pediatric low-grade gliomas, including OPGs, aberrations such as BRAF–KIAA1549 fusion and BRAF V600E mutation constitutively activate the MAPK pathway, driving uncontrolled tumor cell proliferation and contributing to treatment resistance. Persistent MAPK signaling can also alter the tumor microenvironment, enhancing angiogenesis and immune evasion, which may further limit the efficacy of conventional therapies. These molecular insights have provided the rationale for the development of targeted inhibitors of BRAF and MEK in OPG management [20].

**Figure 2:**
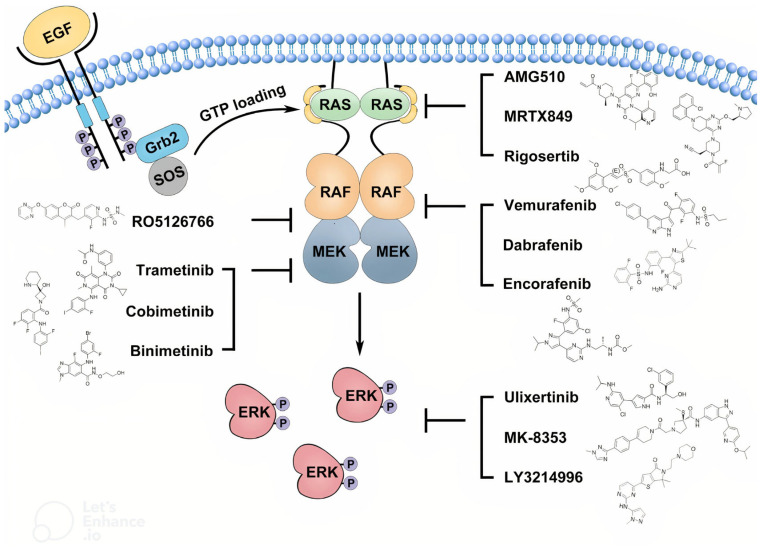
**MAPK pathway and its inhibitors.** Targeting the hyperactive Ras/RAF/MEK/ERK (MAPK) signaling pathway is a promising approach for cancer therapy. This signaling cascade operates downstream of RTKs. When RTKs bind to their ligands, they activate guanine exchange factors, such as Sos proteins, which facilitate the loading of GTP onto Ras GTPases. The GTP-bound Ras GTPases then recruit RAF/MEK heterodimers from the cytosol to the plasma membrane, where RAFs form transient tetramers through side-to-side dimerization. This dimerization activates RAFs, disrupts the RAF/MEK heterodimers, and promotes MEK homodimerization on the RAF dimer surface, leading to MEK activation by RAFs. Once activated, MEKs phosphorylate ERKs, which in turn phosphorylate various downstream effectors. In cancer cells, this pathway can become hyperactive due to mutations in Ras GTPases and BRAF. Such hyperactivity can be targeted with small-molecule inhibitors that specifically inhibit Ras G12C, BRAF(V600E), MEK, and ERK. Abbreviations: MAPK, mitogen-activated protein kinase; RAF, rapidly accelerated fibrosarcoma; RTKs, receptor tyrosine kinases. Copyright/license: This image has been borrowed from Yuan et al. [22], which is an open-access article distributed under the terms and conditions of a Creative Commons license.

Additionally, the overwhelming majority of OPGs are pilocytic astrocytomas which frequently exhibit BRAF alterations. Furthermore, the Pediatric Brain Tumor Consortium (PBTC) data looking at MEKi and OPGs illustrated that even without biopsy, some known pilocytic astrocytomas without a BRAF fusion also had a response. In OPG, the two most common BRAF mutations identified in biopsied cases are the KIAA1549:BRAF fusion and the BRAF V600E point mutation [21]. These mutations activate the MAPK pathway, a key regulator of cell growth and proliferation. Figure 2 provides a simplified illustration of the MAPK pathway and its inhibitors [22].

Interestingly, several MAPK pathway inhibitors have demonstrated activity against OPGs, including selumetinib (MEK1/MEK2 inhibitors), dabrafenib (type I rapidly accelerated fibrosarcoma [RAF] inhibitor) plus trametinib (MEK inhibitor), and tovorafenib (a type II RAF inhibitor) [23,24,25,26,27]. Typically, RAF inhibitors are combined with MEK inhibitors to avoid paradoxical MAPK pathway activation. Table 2 demonstrates a clear overview of the mechanisms, dosages, and side effects for each drug, highlighting their clinical considerations and potential adverse effects.

#### 2.2.2. Evidence Supporting MAPK Pathway Inhibitors

Selumetinib: Selumetinib, a MEK1/MEK2 inhibitor approved for NF–1-associated plexiform neurofibromas, has demonstrated efficacy in both NF–1-associated and sporadic OPG. In a phase II trial by the Pediatric Brain Tumor Consortium, a total of 25 eligible and evaluable patients were included in the study, with a median of 4 prior treatment regimens (ranging from 1 to 11). Among them, 24% (6 patients) achieved a partial response, 56% (14 patients) maintained stable disease, and 20% (5 patients) experienced disease progression during therapy. The median number of treatment cycles administered was 26 (range: 2–26), and over half (14 of 25) completed the full course. The two-year progression-free survival (PFS) rate was 78% (±8.5%). Of the 19 patients assessable for visual acuity, 21% (4 patients) showed improvement, 68% (13 patients) remained stable, and 11% (2 patients) experienced deterioration. Visual field enhancement was observed in 26% (5 patients), while 74% (14 patients) had no change. The most frequently reported adverse effects were mild to moderate (grade 1/2) and included elevated creatine phosphokinase (CPK), anemia, gastrointestinal symptoms (diarrhea, nausea/vomiting), fatigue, headaches, liver enzyme elevations (AST, ALT), hypoalbuminemia, and dermatologic reactions such as rash [23,24].

Tovorafenib: Tovorafenib is a type II RAF inhibitor, which was approved by the FDA in 2024 for children 6 months and older with relapsed/refractory low-grade gliomas harboring BRAF V600E mutations or fusions. In the phase II FIREFLY-1 trial (*n* = 137), tovorafenib achieved an objective response rate of 67% (Response Assessment in Neuro-Oncology criteria) and a median duration of response of 16.6 months. Common adverse events included hair-color changes, elevated creatine phosphokinase, and anemia, with toxicities of grade 3 or higher among 42% of patients [25].

According to the FDA prescribing information, tovorafenib is approved for the treatment of pediatric patients 6 months of age and older with relapsed or refractory low-grade glioma harboring a BRAF fusion or rearrangement, not associated with neurofibromatosis type 1 (NF–1). In vitro studies demonstrated that tovorafenib increased ERK phosphorylation at clinically relevant concentrations in cells with neurofibromatosis type 1 loss-of-function (NF–1-LOF), indicating activation rather than inhibition of the MAP kinase pathway. In an NF–1 genetically engineered mouse model of plexiform neurofibroma lacking BRAF alterations, tovorafenib showed no antitumor activity; moreover, although not statistically significant, tumor volume increased in 2 out of 12 mice (approximately 17%) [26].

Dabrafenib Plus Trametinib: This combination is FDA-approved for BRAF V600E-mutant low-grade gliomas among children 1 year and older. A randomized phase II trial demonstrated its superiority over carboplatin/vincristine, with significant activity in tumors involving the optic pathway or hypothalamus [27].

MAPK pathway inhibitors are emerging as a promising treatment option for optic pathway glioma (OPG), offering the advantage of oral administration over traditional chemotherapy. However, most available studies are phase II trials with small patient cohorts. Therefore, these agents can be considered as potential salvage or second-line therapies, with careful consideration of possible serious adverse effects such as cardiomyopathy and severe skin reactions.

### 2.3. Other Treatment Options

#### 2.3.1. Bevacizumab

Bevacizumab is an anti-VEGF (vascular endothelial growth factor) monoclonal antibody that has a significant role in managing brain tumors such as recurrent high-grade gliomas. Several reports have documented qualitative improvements in vision among children with OPG following bevacizumab-based treatment [28].

Multiple retrospective studies consistently show that bevacizumab, either as a stand-alone treatment or in combination with therapies like irinotecan, can lead to significant visual improvement and radiological responses among 50% to 70% of children with recurrent OPGs, regardless of NF–1 status. Even though bevacizumab shows effectiveness and manageable toxicity, such as systemic hypertension and proteinuria, it has decreased efficacy with prolonged treatment beyond 12 months. Furthermore, over 40% of children experienced recurrence within a few months after discontinuation of bevacizumab, indicating that its effects are not long-lasting [29].

Looking into possible mechanisms of resistance or recurrence, bevacizumab’s anti-angiogenic effect in OPG appears to provide only temporary disease control in many patients, with relapses often occurring within months of discontinuation. At the macro level, this may reflect the drug’s inability to eradicate tumor cells, instead reducing edema and tumor bulk without altering the underlying disease biology. At the micro level, adaptive resistance can occur via activation of alternative pro-angiogenic pathways (e.g., fibroblast growth factor, platelet-derived growth factor), and changes in the tumor microenvironment that restore blood supply despite VEGF blockade [30].

Based on the available evidence, MAPK pathway inhibitors are preferred as second-line systemic therapy for OPG, as their efficacy and safety have been evaluated in at least phase II clinical trials, whereas the evidence supporting bevacizumab in this setting is limited to retrospective studies.

#### 2.3.2. Lenalidomide

Researchers are investigating immune modulators like lenalidomide among pediatric patients whose conditions have worsened despite standard treatments. In a phase II study involving 74 children with pilocytic astrocytoma or OPG, both high-dose and low-dose lenalidomide were tested, resulting in four partial responses in each group. The low-dose treatment was found to be better tolerated by the children [31]. Therefore, the clinical benefit of lenalidomide was not sufficient to support its use as a preferred option for second-line systemic therapy.

#### 2.3.3. Pegylated Interferon α-2b

Pegylated interferon α-2b works by binding to and activating the human type 1 interferon receptors, causing them to dimerize, and this activates the JAK/STAT pathway. Peginterferon α-2b may also activate the nuclear factor κB pathway.

A phase II clinical trial of pegylated interferon α-2B for patients with unresectable juvenile pilocytic astrocytomas and OPGs enrolled nine subjects with a median age of 11 years. The trial demonstrated prolonged stable disease among two patients (75+ and 66+ months) and event-free survival rates of 76.2% at 12 and 24 months, with median event-free survival and overall survival not reached. Side effects were mostly mild (grade 1–2), with no severe drug-related events reported. While the treatment showed safety and potential for delaying disease progression in selected cases, its overall efficacy as a therapeutic option remains uncertain because of the lack of significant tumor responses and small number of enrolled patients [32].

#### 2.3.4. Immunotherapy

**Figure 3:**
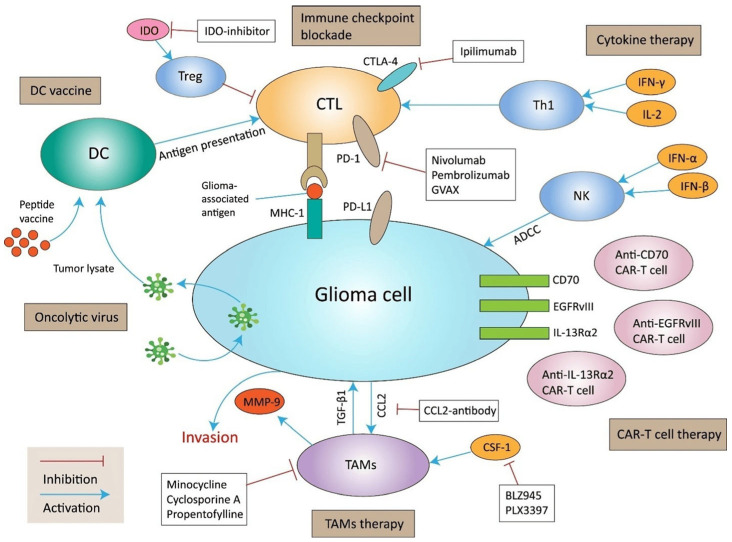
**Current immunotherapy approaches for glioma.** Oncolytic viruses have the ability to lyse glioma cells and subsequently release progeny viruses, which further assist in eradicating residual tumor masses. The tumor lysate released in this process can be identified by DCs, which are integral to DC vaccines. Dendritic cells are highly effective in presenting antigens and can activate CTLs, which then target and eliminate glioma cells. Glioma cells frequently evade immune detection by expressing immune checkpoint ligands such as PD–1, CTLA–4, and IDO. Blocking these checkpoints can significantly disrupt this immune evasion. Cytokines like IFN-γ and IL-2 are capable of activating Th1 cells, thereby enhancing CTL–mediated antitumor responses. Additionally, IFN-α and IFN-β can activate NK cells, which target tumor cells through ADCC. Glioma-associated antigens such as IL–13Rα2, EGFRvIII, and CD70 are expressed on tumor cell surfaces and can be recognized by CTLs with the enhancement of MHC–1 expression. Genetically engineered CAR T cells are targeting these antigens. Moreover, TAMs stimulated by CSF–1 and CCL2 can produce MMP–9 and TGF-β1, promoting glioma cell invasion. Inhibiting these pathways can help reduce glioma cell invasiveness. Abbreviations: ADCC, antibody-dependent cellular cytotoxicity; CAR, chimeric antigen receptor; CTLs, cytotoxic T lymphocytes; DCs, dendritic cells; IDO, indoleamine 2,3-Dioxygenase; NK, natural killer; TAMs, tumor-associated macrophages. Copyright/license: This image has been borrowed from Xu et al. [33], which is an open-access article distributed under the terms and conditions of a CC BY-NC-ND 4.0 license.

To date, no compelling evidence supports immunotherapy as a treatment for OPG. However, there are ongoing clinical trials testing the PD1/PD–L1 inhibitors and CAR T in gliomas [33]. Current immunotherapy approaches for glioma are summarized in Figure 3.

The role of tumor-associated microglia and macrophages (TAMs) in glioma has been increasingly recognized and discussed in literature. TAMs are abundant in the microenvironment of pediatric gliomas and contribute to tumor progression through the secretion of pro-inflammatory cytokines, growth factors, and matrix-remodeling enzymes. These cells can promote glioma cell proliferation, enhance angiogenesis, and suppress anti-tumor immune responses, thereby supporting tumor maintenance and potentially contributing to treatment resistance [34]. Therefore, modulating tumor-associated macrophages (TAMs) has the potential to enhance anti-tumor immune responses and warrants further investigation as a therapeutic strategy.

### 2.4. Ongoing Clinical Trials

These are examples of actively recruiting clinical trials that will assist physicians, patients, and their families in making treatment decisions. These clinical trials are divided into three categories: systemic therapy trials, vision-improvement trials, and CAR T-cell therapy trials, as follows:

#### 2.4.1. Systemic Therapy Trials

Selumetinib: Three phase III clinical trials are investigating selumetinib for low-grade glioma (LGG) at multiple locations in the United States. NCT04166409 compares the efficacy of selumetinib to the standard chemotherapy combination of carboplatin and vincristine (CV) for patients with newly diagnosed LGG that lacks the BRAFV600E mutation and is not linked to NF–1. The trial aims to determine whether selumetinib can provide comparable or improved outcomes, including tumor control and quality of life, compared with CV [35].

NCT04576117 examines the addition of vinblastine to selumetinib in treating recurrent or progressive LGG. This trial seeks to determine whether the combination therapy is more effective than selumetinib alone [36]. Similarly, NCT03871257 evaluates selumetinib vs. CV for treating LGG for patients with NF–1, focusing on tumor control and its potential to improve vision for those patients with OPG [37].

Tovorafenib: Tovorafenib is being compared with chemotherapy in an ongoing two-arm, randomized, open-label, multicenter, global, phase III trial to evaluate the efficacy, safety, and tolerability of tovorafenib monotherapy vs. standard-of-care chemotherapy among patients with pediatric LGG harboring an activating RAF alteration requiring first-line systemic therapy (NCT05566795) [38].

Avutometinib: NCT06104488 is a multicenter phase I dose-escalation study of avutometinib, a RAF/MEK clamp, in pediatric patients with refractory or recurrent solid tumors harboring activating MAPK pathway alterations [39].

#### 2.4.2. Vision-Improvement Trials

The NCT05278715 trial evaluates a modified chemotherapy regimen combining increased-dose carboplatin (220 mg/m^2^), vincristine, and recombinant human endostatin (rh-ES). The trial includes pediatric and adult patients and focuses on visual acuity improvement and response rates over 3 years [40]. Meanwhile, the NCT05733572 trial assesses the safety and efficacy of CHF6467, a recombinant mutated nerve growth factor, for improving visual function among OPG patients. This randomized, double-blind, placebo-controlled study uses CHF6467 eye drops and measures visual-field and visual-evoked potentials over 6 months. The trial targets children (ages 6–17 years) and young adults (ages 18–39 years) [41].

#### 2.4.3. CAR T-Cell Therapy Trials

Three innovative clinical trials are investigating CAR T-cell therapy in gliomas. The first trial (NCT06355908) is a phase I study assessing the safety and feasibility of IL13Ra2-targeted CAR T cells in patients with recurrent or refractory glioma [42]. This trial is focusing on glioma-specific CAR T-cell therapies. Similarly, the second trial (NCT04099797) explores C7R-GD2 CAR T cells for treating GD2- expressing brain tumors, including diffuse intrinsic pontine glioma, high-grade glioma, and medulloblastoma [43]. This phase I study combines i.v. and intracranial infusions to determine the largest safe dose while enhancing T-cell survival with cytokine support.

The third trial (NCT06640582) is a phase I/II study that investigates a combination of tumor-infiltrating lymphocyte therapy with pembrolizumab in advanced brain cancers, including glioblastoma and meningioma [44].

## 3. Conclusions

Managing OPGs is challenging because of their complex location, association with NF-1, and diverse clinical progression. Current treatments, including chemotherapy, targeted therapy, and RT, provide reasonable control in many cases but are limited by issues such as treatment resistance, long-term toxicities, and variability in outcomes. Emerging strategies, including bevacizumab for symptom control and MEK inhibitors for NF–1-associated gliomas, show promise, while immunotherapy and CAR T-cell therapy hold potential to transform the therapeutic landscape. However, the safety and efficacy of these treatments, particularly among pediatric populations, require further investigation.

Future research should focus on developing durable therapies to reduce relapse risk, minimizing toxicities through targeted approaches, and identifying predictive biomarkers for personalized treatment. Integrative care models that address the multifaceted needs of OPG patients and the expansion of clinical trials exploring next-generation therapeutics, such as immune-based therapies and gene-editing techniques, are vital. Collaborations among researchers, clinicians, and patient-advocacy groups will be essential in translating preclinical advancements into effective clinical practices, improving outcomes and quality of life for OPG patients.

## Figures and Tables

**Table 1:** **Carboplatin and vincristine chemotherapy protocol details.** This table summarizes the standard chemotherapy protocol using carboplatin and vincristine, including their mechanisms of action, dosage, administration schedule, and associated side effects. Source: Original table created by the authors based on standard oncology drug information and treatment protocols [12]. Abbreviations: IV—intravenous; mg/m^2^—milligrams per square meter.

Drug	Mechanism of Action	Dosage	Administration Schedule	Common Side Effects	Serious Side Effects
Carboplatin	Alkylating agent that causes DNA cross-linking, leading to apoptosis of cancer cells.	175 mg/m^2^ for 4 consecutive weeks, followed by a 2-week rest period for 12 cycles.	Administered as i.v. infusion over 15–60 min.	Nausea, vomiting, myelosuppression (anemia, neutropenia, thrombocytopenia), and fatigue.	Nephrotoxicity, ototoxicity, hypersensitivity reactions, and severe myelosuppression.
Vincristine	Inhibits microtubule formation in the mitotic spindle, arresting cell division in metaphase.	1.5 mg/m^2^ (maximum dose 2 mg) administered by intravenous bolus weekly concurrent with the carboplatin	Administered as a short i.v. infusion (1–2 min).	Constipation, peripheral neuropathy, jaw pain, and hair loss.	Severe neurotoxicity, including peripheral neuropathy and autonomic dysfunction.

**Table 2:** **MAPK inhibitors-mechanisms of action, dosages, common side effects, and serious side effects.** This table provides an overview of key MAPK pathway inhibitors, summarizing their mechanisms of action, standard dosages, and common versus serious adverse effects relevant to targeted cancer therapy. Source: Original table created by the authors based on prescribing information and published oncology pharmacotherapy references [23,24,25,26,27]. Abbreviations: MAPK—mitogen-activated protein kinase; mg/m^2^—milligrams per square meter.

Drug	Mechanism of Action	Dosage	Common Side Effects	Serious Side Effects
Selumetinib	Inhibits MEK1/MEK2, preventing phosphorylation and activation of ERK1/ERK2 in the MAPK pathway.	25 mg/m^2^ orally 2× daily.	(≥40%): Vomiting, rash (all), abdominal pain, diarrhea, nausea, dry skin, fatigue, musculoskeletal pain, pyrexia, acneiform rash, stomatitis, headache, paronychia, and pruritus	Cardiomyopathy, ocular toxicity, interstitial lung disease, and elevated liver enzymes.
Dabrafenib plus trametinib	Dabrafenib (type I RAF inhibitor) plus trametinib (MEK inhibitor). Both inhibit mutated BRAF V600E kinase, blocking MAPK pathway activation.	Dabrafenib (4.5–5.25 mg/kg/day—divided twice daily) plus trametinib (0.025–0.032 mg/kg/day once daily)	(≥30%): Pyrexia, rash, vomiting, and headache	Severe pyrexia syndromes, cardiomyopathy with decreased left ventricular ejection fraction, ocular toxicities such as uveitis and retinal disorders, cutaneous squamous cell carcinoma, severe skin reactions (e.g., Stevens–Johnson syndrome), hemorrhage, venous thromboembolism, hyperglycemia, interstitial lung disease, and severe hypertension
Tovorafenib (DAY101)	Inhibits RAF dimer-driven MAPK pathway activation.	380 mg/m^2^ orally (max 600) once weekly	(≥30%): Rash, hair color changes, fatigue, viral infection, vomiting, headache, hemorrhage, pyrexia, dry skin, constipation, nausea, dermatitis acneiform, and upper respiratory tract infection	Hemorrhage, severe skin reactions, and hepatotoxicity.

## Data Availability

This review article did not generate any new data. All data discussed in this manuscript are derived from previously published studies, which are cited accordingly in the references section.
